# Psychosocial aspects of infertility and the impact of assisted reproductive techniques - a comprehensive review

**DOI:** 10.5935/1518-0557.20250002

**Published:** 2025

**Authors:** Beatriz Ribeiro Neto, Márcia Barreiro, António Tomé, Emídio Vale-Fernandes

**Affiliations:** 1 ICBAS - School of Medicine and Biomedical Sciences, UMIB - Unit for Multidisciplinary Research in Biomedicine, University of Porto, Porto, Portugal; 2 Centro de Procriação Medicamente Assistida / Banco Público de Gâmetas, Centro Materno-Infantil do Norte Dr. Albino Aroso (CMIN), Centro Hospitalar Universitário de Santo António (CHUdSA), Unidade Local de Saúde de Santo António (ULSSA), Porto, Portugal; 3 Departamento da Mulher e da Medicina Reprodutiva, Centro Materno-Infantil do Norte Dr. Albino Aroso (CMIN), Centro Hospitalar Universitário de Santo António (CHUdSA), Unidade Local de Saúde de Santo António (ULSSA), Porto, Portugal; 4 ITR - Laboratory for Integrative and Translational Research in Population Health, University of Porto, Porto, Portugal

**Keywords:** assisted reproductive technology, infertility, mental health, sociocultural context, parenting

## Abstract

This review examines the impact of Assisted Reproductive Technology (ART) on prospective parents and born children, as well as their parental relationships, considering their emotional and social context. Specific situations are analyzed, such as gynecological pathologies and heterologous treatments, such as the ROPA (Reception of Oocytes from Partner) method, and the use of gestational surrogacy, which have particular psychosocial impacts. Infertility affects approximately one-third of the global population, leading many to seek ART, which have grown in popularity over the decades. In addition to clinical concerns, the psychosocial impact of these techniques has received increasing attention, given the implications for mental health and family relationships. Based on a narrative review methodology, it analyzes experimental and observational studies on the topic, seeking to understand and evaluate the impact of ART techniques. The results highlight the emotional and ethical complexity associated with treatment decisions, from the decision to engage to these treatments to the psychosocial implications of the different available options. Anxiety and depression are common responses to the challenges faced during treatment, affecting both direct beneficiaries and their interpersonal relationships. Gestational surrogacy and heterologous treatments add layers of complexity. Regarding the impact on parental relationships and the development of children conceived through these methods, it is concluded that, despite concerns, children conceived through ART have the potential to grow and thrive as well as naturally conceived ones. This review emphasizes the importance of an inclusive and holistic approach to treating infertility, recognizing that parenthood goes beyond biological conception.

## INTRODUCTION

For prospective parents, conception, pregnancy, childbirth, and the transition to parenthood represent a profoundly significant period ( Crespo & Bestard, 2017 ). In most cases, pregnancy occurs naturally and as planned; however, for about one-third of individuals, this is not the case, and they must come to terms with the possibility of not being able to fulfill this dream ( Gameiro *et al* ., 2014 ).

According to World Health Organization (WHO), infertility is defined as the inability to achieve pregnancy after 12 or more months of regular, unprotected heterosexual intercourse. The American Society for Reproductive Medicine (ASRM) has recently expanded this definition to include the inability to conceive for social reasons, such as being a single parent or part of a same-sex couple. According to WHO, approximately 17.5% of the global population is affected by it, with minor fluctuation across different geographic regions and socioeconomic strata, indicating it as a universal issue ( WHO, 2023 ). About half of the couples facing fertility problems are estimated to need Assisted Reproduc tive Technology (ART) treatments ( Mahany & Smith, 2017 ). These treatments are also inevitable for single people, same-sex couples and couples with a transgender partner without usable gametes. This has led to an increasing use of ART over the last few decades.

Since the birth of the first child conceived via in vitro fertilization in 1978 ( Kamel, 2013 ), millions of children have been born using these techniques, making it pertinent to evaluate their impact.

The medicalization of infertility has sometimes led to an underestimation of its emotional aspects and psychological implications, even though there is significant focus on treatment and scientific research ( Cousineau & Domar, 2007 ). Thus, while initial studies in this area concentrated on improving birth rates, more recent research has shifted towards assessing the psychosocial aspects, as infertility and treatment failures are associated with adverse effects on mental health, such as worse life satisfaction, poor self-esteem, and significant distress ( Hammarberg *et al* ., 2008 ; Johansson *et al* ., 2010 ). Even when treatments are successful and result in pregnancy, barriers like difficult access, which leads to long waiting times, and generally associated high costs, which can lead to financial difficulties, as well as the pressure to which couples are subjected, also affect their well-being and family relationship ( Cousineau & Domar, 2007 ; Silva & Barros, 2012 ).

It is also important to mention heterologous treatments, those involving gamete donation, and the specific psychosocial aspects of such treatments, which have unique characteristics and thus merit analysis. Included in this group of treatments is the ROPA method (Reception of Oocytes from Partner), which is aimed at female couples wishing to have biological children. In this method, one woman provides the eggs, while the other undergoes gestation. This has become an increasingly popular option among female couples desiring a shared biological parenting experience. However, like other ART techniques, the ROPA method also raises ethical, legal, and emotional concerns that deserve careful consideration.

Moreover, within this group of treatments involving donated gametes, in addition to single women and female couples, there are also heterosexual couples for whom the necessity of engaging to donor genetic material generates very specific feelings and attitudes ( Crespo & Bestard, 2017 ).

Surrogacy represents a special case of ART where a woman agrees to gestate and give birth to a baby for another person or couple. This arrangement can be chosen for various reasons, such as conception difficulties, health issues preventing the intended mother from gestating, or in cases of single men, transgender men or male couples. It is a complex issue that raises a multitude of ethical, legal, and social questions, concerning the consent of the gestational carrier, the rights and responsibilities of the intended parents, and the emotional and physical well-being of all involved parties, especially the child to be born and the children already born of the gestational carrier.

Another specific technique that presents unique challenges and concerns is posthumous-assisted reproduction (PAR), a form of ART that includes the use of sperm, eggs, or embryos that have been cryopreserved before or immediately following the death of an individual ( Lawson *et al* ., 2016 ). It is an important topic to address due to its profound implications for both surviving partners and the children born from such procedures. The use of reproductive material after one partner’s death introduces unique emotional and ethical challenges.

This review aims to assess the psychological and social impact after engaging to ART techniques on the recipients and born children, as well as on their parental relationship. Gaining a deeper understanding of these aspects will be beneficial for a more tailored and individualized approach by healthcare professionals dealing with infertile couples or women searching these techniques, ultimately leading to improved medical care ( Crespo & Bestard, 2017 ).

## METHODS

**Figure 1:**
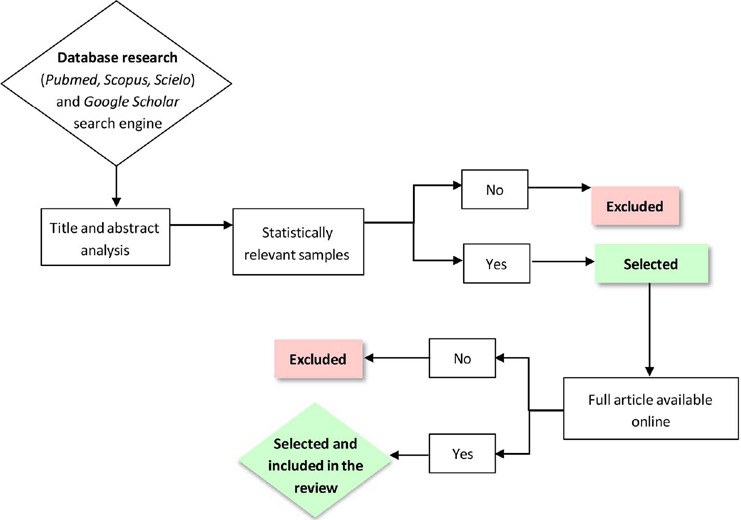
Flowchart of article search and validation mode.

The methodology for this narrative review is outlined in Figure 1 , and it involves bibliographic research conducted in medical and scientific databases such as PubMed, Scopus, Scielo, and the Google Scholar search engine. This review includes articles published between 1993 and 2023 that have experimental or observational designs, conducted on humans, about the psychosocial impact of infertility and ART. The research strategy was developed and executed between September 2023 and February 2024. The search was designed using the following keywords: “reproductive techniques, assisted,” “in vitro fertilization,” “pregnancy,” “parenting,” “psychology,” “psychological adaptation,” “infant behavior,” and “parent–child relations.” Various types of studies were included.

Articles were selected based on specific criteria, namely, if the samples were statistically relevant and if the psychological and social impact of the techniques was evaluated, not just the clinical impact.

Studies were excluded if the full article was not available online. Only studies available in English and Portuguese were considered.

## THE IMPACT OF ASSISTED REPRODUCTIVE TECHNIQUES (ART) ON BENEFICIARIES

The context surrounding individuals who engage to ART is notably diverse, encompassing a socio-demographically heterogeneous population with distinct characteristics when compared to couples who conceive naturally ( Hammarberg *et al* ., 2008 ). Such differences include a higher average age at conception—due to the delays caused by the diagnosis of infertility and numerous treatment attempts—a longer relationship duration with their current partner, higher prevalence of multiple pregnancies, and specific reproductive histories, such as fewer previous pregnancies and more miscarriages ( Hammarberg et al., 2008 ). These unique conditions are difficult to replicate in a control group, therefore requiring careful consideration when analyzing the following aspects. A summary of the literature’s findings regarding the psychosocial impact of ART on beneficiaries is provided in Table 1 .

**Table 1:** Psychosocial Impact of Assisted Reproductive Techniques (ART) on Beneficiaries.

Author, year country	Questions/groups analyzed	Main findings
Tendais & Figueiredo, 2016 Portugal	Comparison of levels of anxiety and depression in pregnancy and postpartum in couples undergoing fertility treatment	Parents after fertility treatments showed higher levels of anxiety, and these levels increased even more in the postpartum period. Male subjects showed no statistically significant differences.
Hammarberg *et al* ., 2008	Psychosocial aspects of pregnancy, birth and the first year postpartum after conception by ART	Individuals who engage to ART have similar levels of depression compared to the rest of the population. As far as anxiety is concerned, the evidence is equivocal, with general anxiety levels being similar to those who conceive naturally. However specific anxieties, about pregnancy safety and fetal health, are higher in women who engage to ART, especially those who have experienced more treatment failures and greater infertility-related stress.
Verhaak *et al* ., 2007b Netherlands	Evaluation of long-term psychological adaptation to fertility treatments	Treatments that resulted in pregnancy led to better emotional adjustment. In addition, most of the women who did not become pregnant adjusted well psychologically, returning to baseline levels after an initial increase in anxiety. This positive adaptation was related to the development of new life goals.
Cox *et al* ., 2006 United Kingdom	Investigation of self-esteem in pregnancy after infertility and its correlation with anxiety	Women who underwent IVF showed similar levels of selfesteem to those who conceived naturally.
Holter *et al* ., 2006 Sweden	Evaluation of couples' shortterm emotional response to their first IVF treatment	Those who don't achieve pregnancy report worse emotional state, while those who do achieve pregnancy feel better than they did at the start of the treatment. This effect is especially noticeable in women, but male partners react with the same emotional pattern. Most couples reported an improvement in their marital relationship during treatment.
Crespo & Bestard, 2017 Spain	Evaluation of the transition period between infertility and parenthood	Couples who undergo ART show greater anxiety. This is attributed to a complexity of reasons, including the period of infertility and the number of treatment attempts, the confrontation with the unexpected need for donor gametes, the risk of medical complications, and the relationship with the partner and/or social support.
Dann, 2014 New Zealand	Evaluation of the experience of first-time pregnancy and motherhood after repeated fertility treatments (≥3)	Infertility is an experience with major psychosocial impact on women who go through it, and it does not disappear even after a confirmed pregnancy. These women tend to develop better coping mechanisms, and the experience of repeated failed treatments and pregnancy losses has an impact on how they welcome pregnancy and make the transition to motherhood.
Peterson *et al* ., 2008 Denmark	Investigating the impact of the partner’s coping strategies on the experience of infertility	The way an individual deals with infertility plays a fundamental role in the way their partner deals with it. The use of active avoidance is related to an increase in personal, marital and social anxiety for both men and women. When a woman uses the active confrontation mechanism, marital stress increases.
Brandão *et al* ., 2023	Review of the impact of the ROPA method (Receipt of Oocytes from the Partner)	The ROPA method’s impact on the parenting relationship is still uncertain and not studied enough.
Voultsos *et al* ., 2019 , Greece	Evaluation of the experience and attitude towards motherhood in lesbian women	Lesbian women tend to desire having biological children, as they feel that pregnancy would bring them a sense of fulfillment. They also report the negative impact of social oppression and prejudice on their reproductive autonomy.
Pelka, 2009 United States of America	Evaluation of feelings of jealousy in lesbian couples in cases of shared maternity	Feelings of jealousy are reported in couples of women who engage to single-parent methods, related to factors such as both wanting to be the biological mother, perception of unequal biological ties to the child, and infertility.
Söderström- Anttila *et al* ., 2016	Review of the outcomes of surrogacy for mothers, children, and the motherchild relationship	Most surrogate pregnancies are successful, and most gestational carriers are motivated and have no difficulty separating from their newborn. The perinatal outcome for the children is comparable to other children born after ART.

The literature primarily assesses the impact of ART on beneficiaries—ranging from infertile couples to single women or female couples—through indicators such as depressive symptoms, anxiety, and self- esteem, often yielding inconclusive or contradictory results ( Hammarberg *et al* ., 2008 ). However, a clear distinction is observed in the emotional responses based on whether the treatments result in pregnancy.

While some studies report higher levels of anxiety and depressive symptoms among women who achieve a viable pregnancy through ART compared to those who conceive naturally, other studies, do not show a higher rate of distress in ART pregnancies ( Capuzzi *et al* ., 2020 ; Tendais & Figueiredo, 2016 ). This indicates a need for further research to understand the varying psychological responses among ART patients ( Tendais & Figueiredo, 2016 ). Some studies show that this anxiety particularly relates to pregnancy- specific concerns, such as fetal health, delivery complications, and postnatal separation anxiety, peaking in the third trimester and the first postpartum week ( Hammarberg *et al* ., 2008 ). These worries are more prevalent among women who have undergone longer treatment periods or experienced significant infertility-related stress ( Hammarberg *et al* ., 2008 ; Tendais & Figueiredo, 2016 ). Some studies suggest that women who achieve pregnancy through ART may report better emotional well-being in the long term compared to those whose treatments are not successful. However, numerous studies have also found that childless individuals after infertility treatments report similar levels of emotional well-being as those who had children. It is important to note that all studies in this area are correlational and cannot definitively attribute longterm emotional well-being to having or not having children through fertility treatment ( Verhaak *et al* ., 2007a ). The psychological well-being of male partners shows less difference, suggesting potential methodological influences rather than genuine disparities in response, as there is a tendency for women to express their feelings more readily to strangers than men ( Petok, 2015 ).

Following unsuccessful treatments, initial increases in anxiety and depression are reported, with some studies suggesting a return to baseline levels within 3 to 5 years ( Verhaak *et al* ., 2007a ). However, more recent literature indicates a more complex picture, with some findings suggesting that the highest levels of distress are evident between 4 and 7 years of unsuccessful treatment. Additionally, ongoing treatment and eventual treatment discontinuation have been associated with lower quality-of-life, as measured by FertiQOL ( Verhaak *et al* ., 2007b ). Regarding individuals’ self-esteem, the literature does not report any significant differences between individuals who underwent ART techniques and those who conceived spontaneously ( Cox *et al* ., 2006 ). Interestingly, repeated treatment failures appear to have a diminishing psychological impact over time, suggesting an adjustment process ( Cox *et al* ., 2006 ). This pattern is similarly observed in male partners, although their overall psychological impact is generally reported as less significant ( Holter *et al* ., 2006 ). These findings highlight the varied and evolving nature of psychological responses to ART, underscoring the need for individualized psychological support throughout the treatment journey.

Several factors exacerbate anxiety levels among ART beneficiaries, including the uncontrollable element of parental age, which becomes particularly critical given the extended waiting periods associated with ART, especially in public health systems. Advanced maternal age is linked to increased obstetric risks, thereby heightening anxiety levels, even when pregnancies are successful ( Crespo & Bestard, 2017 ). Additionally, previous treatment failures and history of pregnancy loss contribute to poorer emotional states, framing pregnancy as a highly stressful period filled with uncertainty ( Dann, 2014 ).

Conversely, positive emotional outcomes are associated with a strong emotional support network, available counseling services and the individual’s personality ( Verhaak *et al* ., 2007a ). Regular medical follow-up, in the case of successful treatments, and setting new life goals, after unsuccessful treatments, also contribute to better emotional recovery ( Crespo & Bestard, 2017 ; Verhaak *et al* ., 2007a ).

Results obtained through these techniques depend on various factors, not only epidemiological factors like the woman’s age and causes of infertility and clinical practice adopted but also the social context in which the techniques are applied, although the role played by each remains uncertain ( Castilla *et al* ., 2009 ). The need for ART is universal, as infertility is widespread globally; however, differences in social context and access to these techniques vary greatly from country to country ( Ferraretti, 2014 ). Therefore, structural factors, such as socioeconomic status, healthcare policies, and access to supportive services, significantly impact individuals’ experiences and emotional well-being. Access to ART can be profoundly influenced by financial resources, insurance coverage, and local availability of treatment centers. For instance, the cost of ART procedures often requires substantial financial investment, which can be prohibitive for many, leading to disparities in treatment accessibility. Moreover, insurance coverage for ART varies widely across different countries and regions, with some offering comprehensive coverage while others have none of that help. These structural factors can exacerbate emotional distress among individuals and couples undergoing ART, especially when facing financial strain or navigating complex healthcare systems.

Additionally, sociocultural factors, such as societal attitudes towards infertility and ART, can influence individuals’ experiences, potentially affecting their willingness to seek treatment and the support they receive. One study shows that those with higher levels of education, employment, higher incomes, private health insurance, higher social support, stronger religious beliefs, and higher spiritual well-being reported better mental health outcomes ( Bagade *et al* ., 2023 ).

Recent research highlights that having certain types of employment may also impact the ability to meet IVF treatment demands. Furthermore, employment types that allow for flexible scheduling or time off can significantly affect the ability to pursue and continue fertility treatments (Wilkinson *et al* ., 2023).

Another important factor in this regard is each country’s legislation on assisted reproduction, encompassing aspects like restrictions on the number of embryos that can be transferred or the intended purpose for frozen embryos ( Castilla *et al* ., 2009 ). Other relevant factors in the social context include competition between clinics and infertility care coverage, which determines treatment accessibility ( Castilla *et al* ., 2009 ).

Although each country’s legislation generally affects both public and private clinics, primarily regarding the maximum number of embryos to transfer per treatment cycle, differences may arise concerning the clinical practice applied in each case, which is crucial to the outcome ( Castilla *et al* ., 2009 ). For example, while public clinics in many countries have waiting lists and restrictions on the age of accepted patients and the number of cycles per patient, private clinics have complete freedom in these aspects. Thus, patients often choose to undergo initial cycles in private clinics while waiting in the public sector ( Peterson *et al* ., 2008 ). These aspects put immense pressure on individuals engaging to these techniques, both because they feel in a “race against time” due to the constraints imposed by public hospital rules, especially regarding age limits and waiting lists, and because of the economic burdens imposed if they do not achieve pregnancy within the number of cycles they can afford ( Golombok *et al* ., 2002 ). In this sense, couples from Canada and the United States, where fertility treatments are not (or were not at the time of the study) covered by public health services, reported financial issues as the main reasons for discontinuing treatments, alongside psychosocial distress ( Golombok *et al* ., 2002 ).

The experience of infertility and undergoing ART treatments is often a shared journey for couples, making it crucial to examine the interaction and relationship dynamics, as well as how individual reactions impact their partner’s adjustment ( Peterson et al., 2006a ; b ). There is literature showing that most couples report no negative impact on their relationship regardless of the treatment outcome, often emerging with strengthened bonds, making an effort to be together and support each other during this process ( Holter *et al* ., 2006 ). This resilience might be attributed to a selection effect, where couples with more stable relationships and equal commitment towards parenthood are more likely to pursue and persist with ART treatments ( Hammarberg *et al* ., 2008 ). In fact, a study shows that by the time children reach the age of 11-12, more than 90% of couples were still married ( Peterson et al., 2006a ; b ).

The interdisciplinary teams and professionals involved in ART should prioritize psychoeducation, ensuring that partners are included in the process ( Reisi *et al* ., 2024 ). It is essential to acknowledge and actively engage men during infertility assessments ( Shorey & Chan, 2020 ). Recognizing paternal vulnerabilities and needs requires a shift in the mindset of professionals within the broader perinatal context (Angelelli, Forthcoming; Schöch *et al* ., 2024 ). According to psychodynamic theory, the therapeutic setting encompasses the spatial, temporal, contextual, and regulatory frameworks that facilitate a secure interaction between the patient and the therapist (Angelelli, Forthcoming).

Analyzing how one partner’s coping method impacts the other and their relationship, the literature focuses on three specific methods: active avoidance, active confrontation, and passive avoidance ( Peterson et al., 2006a ; b ). Individuals practicing active avoidance strategies avoid being around pregnant women or situations that remind them of infertility, turning to other external activities like work, distancing themselves from the reality of infertility ( Peterson et al., 2006a ; b ). Studies show that this adaptation method is associated with greater personal and social suffering, negatively affecting the partner, even if they do not practice this strategy ( Klemetti *et al* ., 2004 ; B. D. Peterson *et al* ., 2006). On the other hand, active confrontation involves individuals actively expressing their feelings about infertility, seeking help and support from others. Results for this method are contradictory. While some data reveal that incongruence in reactions within a couple, where one partner is more reserved and the other openly expresses feelings about infertility and seeks advice from others, has a negative effect on the male partner, increasing his anxiety levels, other studies suggest that men and women are more disturbed when the woman keeps her feelings to herself while the man is more open and expresses them ( Peterson et al., 2006a ; b ). Despite this contradiction, it is relevant to highlight how incongruence and the choice of different approaches within the same couple consistently contribute to negative effects for at least one partner, leading to marital conflict ( Gameiro *et al* ., 2012 ; B. D. Peterson *et al* ., 2006). Regarding passive avoidance, individuals in this group wait for a miracle or feel that all they can do is wait, and no significant psychosocial impact was observed in this group ( Peterson et al., 2006a ; b ).

## SPECIAL SITUATIONS

### Special Medical Situations

There are some medical conditions that predispose infertility and may be the reason for engaging to ART. Thus, it is relevant to analyze these cases and their psychosocial impact. The ones selected were polycystic ovary syndrome (PCOS), endometriosis, and premature ovarian insufficiency, for their high prevalence, strong association with infertility, and extensive study.

PCOS is one of the most common endocrine disorders in women of childbearing age and a significant public health problem. This condition can cause a variety of longterm health problems, impacting the physical and mental well-being of women, and is a major cause of infertility. The literature suggests a high prevalence of symptoms and psychiatric pathology, such as depression, anxiety, bipolar disorder, obsessive-compulsive disorder, and somatization, recently being associated with a higher risk of suicide attempts compared to the rest of the population ( Brutocao *et al* ., 2018 ; Hsu *et al* ., 2024 ).

Endometriosis is also a frequent gynecological pathology associated with infertility, affecting about 10% of women of reproductive age. Like PCOS, it has been associated with depressive and anxious symptoms and a lower quality-of-life, with intense pelvic pain, as well as concerns regarding sexual dysfunction and infertility, seeming to underlie this depressed mood ( La Rosa *et al* ., 2020 ; Laganà *et al* ., 2017 ; Roomaney & Mitchell, 2022 ).

Similarly to the cases presented previously, patients with premature ovarian insufficiency experience the loss of reproductive function, in addition to the physical symptoms of the disease, and both factors can impact their quality-of-life ( van Zwol-Janssens *et al* ., 2024 ). Once again, the literature points to a reduction in quality-of-life indicators, with an increased risk of depression and anxiety ( van Zwol-Janssens *et al* ., 2024 ; Xi *et al* ., 2023 ).

### Heterologous Treatments

Although ART is widely used globally, it is not universally accepted. For instance, devout Catholics and adherents of other religious groups often oppose to the use of assisted methods that involve fertilization outside the context of intercourse, such as IVF and IUI ( Radkowska-Walkowicz, 2018 ). These ethical and religious considerations raise doubts for some couples, especially when it is necessary to engage to gamete or embryo donation.

While ART has gained widespread acceptance, the same cannot be said when it comes to gamete or embryo donation. This raises concerns and uncertainties for some couples who may be considering these options ( Crespo & Bestard, 2017 ). For some people, it is important that the child born is genetically related to them, as physical similarities tend to promote the relational aspect of kinship, strengthen moral bonds, and constitute an important part of a person’s identity ( Kantsa *et al* ., 2015 ). Opting for heterologous treatments is not always easy, nor is it the one considered at the beginning of the process, except in cases of single women or same-sex couples, in which this is the only option ( Crespo & Bestard, 2017 ). Studies reveal that in heterosexual couples seeking treatment for infertility, engaging to heterologous treatments occurs for three main reasons: failure of previous treatments, advanced age, and/or a medical history of some pathology, namely genetic, which prevents or contraindicates the use of their own gametes ( Crespo & Bestard, 2017 ). This need to engage to donor gametes proves to be a challenge for some couples, especially because it is an alternative that most of them did not anticipate, and it is a confrontation with the reality of not being able to conceive naturally, which often affects individuals’ self-esteem, as they feel less capable, besides increasing the stress and anxiety associated with this situation ( Crespo & Bestard, 2017 ). Additionally, individuals express fear of their peers’ reaction and anxiety when anticipating the moment they will tell the child that they were the result of gamete donation ( Crespo & Bestard, 2017 ).

Literature shows that these concerns are accentuated when individuals choose embryo donation. This situation poses unique challenges as it implies that none of the genetic material comes from the future parents of the child. This lack of biological connection may raise some questions and insecurities, such as concerns about the child’s sense of identity, fears of not bonding as strongly with the child, and worries about potential social stigma. Yet, studies regarding its social impact are scarce and mostly theoretical, not actually expressing opinions or feelings of individuals going through this process. Still, raised concerns revolve around this absence of genetic connection between the child and the parents and the complex family structures that this method can originate, including donors, receiving parents, children from both parties, and other family members. In this sense, questions also arise about the interaction between the two families (if such relationship exists) and how the children from both parties will react when learning about the existence of biological siblings in another family ( Jadva & Imrie, 2023 ; Samorinha *et al* ., 2020 ).

Recent literature highlights the potential psychosocial and health impacts on gamete donors, especially given the increasing likelihood that donors can be identifiable despite intentions for anonymity. Careful psychological screening and psychoeducation of donors are warranted. ASRM and ESHRE guidelines emphasize the need for comprehensive psychosocial assessments to address the emotional impacts on donors and ensure their well-being throughout the donation process. Similarly, it is crucial to conduct psychological screening and implications counseling for gestational carriers, their partners, and recipient parents. This practice, endorsed by ASRM and ESHRE, significantly impacts the emotional well-being of all parties involved in a surrogacy arrangement, helping to navigate the complex emotional and psychological dynamics inherent in these processes ( Freeman, 2015 ).

### ROPA Method (Reception of Oocytes from the Partner)

Another case that should be analyzed for its specificities is shared motherhood in female couples and the use of the ROPA method. This is an ART for female couples in which one provides the oocyte and the other receives the embryo and carries it during pregnancy ( Bodri *et al* ., 2018 ). This type of treatment is not legal in all countries, but by the end of 2021, it could be used without restrictions in 13 European countries ( Brandão, 2022 ). Other options for these couples include artificial insemination or IVF with donor sperm, where both women are legally mothers, but from a biological point of view, these are monoparental methods, as only one of them undergoes all procedures. Thus, the ROPA method presents itself as a biparental method, as it allows both women to have an active role in the conception of the newborn ( Brandão *et al* ., 2023 ).

The reasons behind the choice of this method include not only allowing the couple to share biological motherhood but also clinical and obstetric reasons, such as ovulatory or uterine dysfunction in one of the women, or in cases of transgender individuals who underwent gender affirming surgery after fertility preservation ( Eyler *et al* ., 2014 ).

Studies on this method, despite its increasing use, are limited and often rely on expert opinions or perspectives of women yet to undergo treatment, leading to sparse and unclear findings ( Brandão & Ceschin, 2023 ; Brandão *et al* ., 2022 ). Theoretically, this method may enhance the mother-child bond by allowing both partners to assume maternal roles, potentially strengthening the couple’s relationship ( Roth, 2017 ; Voultsos *et al* ., 2019 ). Moreover, this method allows the burden of ART to be shared by both parents, which does not happen in cases of heterosexual couples, where the woman is generally subjected to more procedures and more invasive ones ( Roth, 2017 ). With the ROPA methos, the couple shares this burden at the physical, mental, and emotional levels, with none of the women having to take on the role of a mere spectator ( Brandão & Ceschin, 2023 ). Both partners are actively involved, mitigating feelings of exclusion or jealousy often reported in monoparental treatments among female couples ( Pelka, 2009 ).

### Surrogacy

Another situation that must be considered is the use of surrogacy. This involves a woman carrying a child for another individual or couple, with no intention of parenting the child herself, utilizing either donor gametes or those of the prospective parent ( Shenfield *et al* ., 2005 ). The main indication for engaging to this technique is the congenital or acquired absence of a functional uterus; however, it is also employed in instances of maternal medical conditions that may pose risks during pregnancy and in the biological impossibility of conceiving or bearing a child, which applies to male couples, single men, or transgender individuals ( Blake *et al* ., 2017 ; Brinsden, 2003 ). Surrogacy raises various ethical questions and is not legal in some European countries ( Brunet *et al* ., 2012 ).

Regarding the psychological impact on future parents and their marital relationship, no significant differences were detected between these and those engaging to other forms of ART ( Söderström-Anttila *et al* ., 2016 ). Instances have been documented where parents have rejected children post-gestation, such as the case of baby Gammy. His parents left him with the gestational carrier after birth due to his Down syndrome condition, opting to only take his healthy twin sister to Australia, their country of residence ( Schover, 2014 ). This case draws attention to situations in which the fetus presents some disability or malformation, and future parents may not accept the child and even blame the gestational carrier, creating a complex problem. Thus, these peculiarities must be ensured in the surrogacy agreement, so that parents cannot abandon the child with the gestational carrier. Fortunately, these cases are rare, and the risk of parents not accepting children after gestation in Western countries is currently low ( Brinsden, 2003 ). Couples who, after a process of examinations and counseling, remain committed to this complex and time-consuming process, are generally very motivated to become parents. Nonetheless, the importance of pre-treatment counseling and monitoring during pregnancy is emphasized ( Söderström-Anttila *et al* ., 2016 ).

It is relevant to address the existence or not of compensation for the gestational carrier and how this compensation occurs. Surrogacy can be designated as commercial or altruistic, depending on whether the gestational carrier receives monetary compensation for the pregnancy, with legislation differing in different countries ( Schenker & Cain, 1999 ). In commercial surrogacy, the gestational carrier is usually recruited through an agency (common in the United States), reimbursed for medical expenses, and paid for her gestational services ( Schenker & Cain, 1999 ). This, being considered a business, ends up being associated with greater pressure exerted on agencies, if that was the case, and on the gestational carrier herself to deliver a “valid end product” that is, a healthy newborn, to their “clients,” the future parents ( Sydsjö *et al* ., 2019 ). Moreover, poverty, illiteracy, and lack of economic development in their country can lead to disadvantaged women becoming gestational carriers for the financial reward, increasing the possibility of exploitation by the cross-border reproductive care industry and by future parents ( Sydsjö *et al* ., 2019 ). On the other hand, in altruistic surrogacy, the gestational carrier is found through friends, family, acquaintances, or advertising and may be reimbursed for health expenses directly related to pregnancy and for lost wages due to this ( Schenker & Cain, 1999 ). In these cases, as the gestational carriers are often close to the family, they will probably have contact with the child after birth and during their upbringing, which can have a significant psychological impact on these women ( Schenker & Cain, 1999 ). Gestational carriers are compensated for however far along in the treatment/ pregnancy process they go; and there is pressure on any gestational carrier (compensated or altruistic) to carry a fetus to term ( Schenker & Cain, 1999 ; Sydsjö *et al* ., 2019 ).

Since surrogacy is illegal in much of the Western countries, infertile couples seek commercial surrogacy agreements elsewhere, such as Russia, Ukraine, and India, where these treatments are available ( Brandão & Garrido, 2022 ; Söderström-Anttila *et al* ., 2016 ). It was estimated that more than 25,000 children were born through gestational carriers in India, more than half of which are Westerners ( Söderström-Anttila *et al* ., 2016 ). Cross-border surrogacy is an activity that raises ethical and legal issues, putting both parents and the gestational carrier at risk and may leave the child vulnerable in various aspects (Söderström- Anttila *et al* ., 2016). However, perhaps contrary to what would be expected, the literature reports high satisfaction levels of future parents when engaging to treatments outside their country, stating that they feel more in control of the treatment, being able to be actively involved in decisions regarding the protocol, donors, time, as well as establishing a closer relationship with the clinician ( Jackson *et al* ., 2017 ). This may reflect a disparity between the opinion of healthcare professionals, who tend to believe that the parents’ focus is clinical success and treatment costs, while they often value personal connections more. Regarding legal problems, most parents are unconcerned about the possibility of lack of legal parenthood, valuing only the child’s health and well-being and the fact that they can be together ( Jackson *et al* ., 2017 ). However, these legal issues and having to go through a bureaucratic process to ensure legal parenthood in their country of origin may raise psychosocial problems, both during pregnancy and during the child’s entire childhood.

Many studies report positive and fulfilling relationships between gestational carriers and the intended parents. These relationships are often characterized by clear communication, mutual respect, and emotional support. Additionally, research indicates that most gestational carriers do not experience negative psychological outcomes; they often express satisfaction with their role and a sense of fulfillment from helping others achieve parenthood ( Greenfeld, 2015 ; Söderström-Anttila *et al* ., 2016 ).

Studies also indicate low levels of parental stress, with both heterosexual and homosexual couples being open about engaging to surrogacy, even in countries where it is not legally permitted ( Sydsjö *et al* ., 2019 ). Generally, individuals are not confronted with hostility or questioned when they mention engaging to surrogacy, receiving support from family, friends, healthcare professionals, and the child’s school environment ( Jackson *et al* ., 2017 ). However, some heterosexual couples choose not to disclose their child’s surrogacy conception due to fear of stigma, particularly in countries where surrogacy is prohibited ( Jackson *et al* ., 2017 ).

On the other hand, homosexual couples tend to be more open about their child’s conception method ( Jackson *et al* ., 2017 ). Despite no significant differences being noted between heterosexual and homosexual couples regarding the time needed to ensure legal custody of the child and their nationality, some same-sex couples describe negative experiences with individuals they had to contact during this process ( Brandão & Garrido, 2022 ).

### Posthumous assisted reproduction

The literature on PAR is limited, but existing studies indicate that the implications for the well-being of all parties involved can be profound. The field of fertility preservation (FP) has evolved significantly in recent years, offering new possibilities for individuals with life-threatening illnesses, providing patients with a chance to experience genetic parenthood posthumously ( Polyakov & Rozen, 2023 ). There are two groups of people involved in this process that should be analyzed separately: the individual with the life-threatening illness and the partner, who will be the single living parent of the child. While both are clearly important, the third critical stakeholder is the child born through a method of conception that guarantees that they will have an entire life with no relationship with one of their genetic parents.

For individuals facing the uncertainty of a poor prognosis, PAR can offer solace and a sense of legacy in the knowledge that should their partner conceive using their genetic material, together they will create new life ( Jones *et al* ., 2023 ). It grants their imagination of parenthood and their role in the lives of generations beyond even after the loss.

For the surviving partners, this may provide some consolation with a biological part of their deceased loved one being carried forward in the world ( Polyakov & Rozen, 2023 ). On the other hand, this can also cause discomfort and conflicting thoughts, since he or she might feel obligated to try again which may not always be in their best interests or wish ( Polyakov & Rozen, 2023 ).

The practice of posthumous reproduction is governed by diverse legal and regulatory frameworks worldwide, which greatly influence its feasibility and ethical considerations ( Lawrence *et al* ., 2022 ).

## THE IMPACT OF ASSISTED REPRODUCTIVE TECHNIQUES (ART) ON PARENTING

The transition to parenthood is a significant moment in an individual’s life, involving major changes from psychological, sociocultural, and biological perspectives ( Agostini *et al* ., 2020 ). Concerns are raised that the anxiety and depression experienced by prospective parents during the period of infertility and ART treatments may have a negative impact on the subsequent parenting relationship ( Covington & Burns, 2006 ). Table 2 reflects a summary of the literature’s findings regarding the psychosocial impact of ART on parenting.

**Table 2:** Psychosocial Impact of Assisted Reproductive Techniques (ART) on Parenting.

Author, year country	Questions/groups analyzed	Main findings
Agostini *et al* ., 2020 Italy	Compare interaction styles between parents and children post ART and those that conceived spontaneously e studies the variability between specific ART treatments	The ART techniques seem to partially influence the constructions of parent-children’s interactions, with the ART beneficiaries being described as more frequently misfit and with interactive patterns of risk. In addition, specific clinical variables, such as the treatment being ICSI, or the existence of previous treatments, present themselves as additional stress factors which raise the tension already experienced by couples in the transition to parenthood.
Gibson & McMahon, 2004	Analyze parenthood after fertility treatments	The fertility treatments don’t seem to have adverse effects on parenthood, as the differences found in relation to the couples that conceive spontaneously don’t indicate clinical problems or have implications on the psychosocial development of the children, having more to do with preoccupations which should be framed in the specific context of anxiety and added pressure in which this pregnancy and birth occur.
Tallandini *et al* ., 2016	Study whether the genetic connection and/or age of the child influences the will of the parents to talk to it about the way it was conceived	Few parents that engaged to ART opt to tell their children they engaged to these techniques, with the type of treatment (homologous vs heterologous) being the main variable. Those that engage to genetic material from a donor present more reluctance to reveal to their children, this difficulty being associated to the absence of biological connection.
Applegarth *et al* ., 2016 United States of America	Analyze if the parents that engage to oocyte donation have the intention of revealing this to their children and if they carry out their intention	43% of the population of the study revealed the use of oocytes from a donor to their children as they intended, 39% still wanted to reveal, 9% were still uncertain and 9% pretended to never reveal. The main reasons behind the wish to reveal were to promote an environment of truth and honesty in the family. The decision to postpone the reveal associated with higher levels of anxiety in the parents.
MacCallum *et al* ., 2008 United Kingdom	Compare the attitude of the parents relatively to the gamete donor with those that engage to adoption	The individuals that engage to gametes from a donor have little information about the donors (only physical characteristics), and they think and talk less about them than those that engage to adoption talk and think about the biological families. Adoptive parents have a bigger tendency to reveal their origin to children than parents that engage to heterologous treatments.
Vitule *et al.* , 2015 Brazil	Analyze the ideas of same sex couples about the use of ART techniques in the implementation of the parenthood project	The biological bonds are preponderant in the discourse of women, with a tendency to desire engage to ART, above all the ROPA method. Men, even when they show the desire to have a genetically similar child, tend to opt for adoption, rendering as the main motive the fear of the bond that might be established, through gestation, between the gestational carrier and the child.
Golombok *et al* ., 2002 Europe	Analyze the transition to adolescence in the children born out of ART	Families that engaged to ART are similar to rest in most quality of the parenthood relationship parameters. The differences noted mostly reflect a better family functioning in the families post ART, with the exception of an exaggerated involvement of the parents in a small percentage.

Research indicates that the use of ART generally has minimal direct effects on parenting and parent-child relationships. Any differences identified are often influenced by specific methodological factors rather than indicating clinical issues or significant implications for children’s psychosocial development and should be understood within the specific context in which this pregnancy and birth occur, often a lengthy and challenging journey ( Agostini *et al* ., 2020 ; Gibson & McMahon, 2004 ). Challenges typically arise during pregnancy and the early postnatal period, possibly due to idealized perceptions of parenthood and the complex journey of ART conception, as well as parental perception of the child as “special” ( Agostini et *al* ., 2020 ; Hammarberg *et al* ., 2008 ). While some studies report a more protective and affectionate attitude among parents using ART ( Covington & Burns, 2006 ; Brennan D. Peterson *et al* ., 2006), others suggest potential concerns regarding maladjustment and less empathetic behavior ( Agostini *et al* ., 2020 ). Further investigation is needed to understand the full implications of ART on parenting dynamics, as existing data do not provide a clear consensus.

Analyzing these data, it is relevant to take into account the presence of social protective factors, which are generally more prevalent in individuals using ART compared to those who conceive spontaneously. These factors include older parental age, higher level of education, greater financial stability, more satisfactory marital relationships, greater adaptability, and problem-solving skills, which may minimize adverse parental outcomes ( Covington & Burns, 2006 ).

Furthermore, the literature reveals that the parenting relationship differs depending on the sociocultural environment in which the family is embedded, with authors of one study identifying greater difficulties in parental adjustment in Eastern European families compared to Western European ones ( Cook *et al* ., 1997 ).

The moment of disclosure, when parents tell their children that they were born through ART, is also recognized as crucial in this process, with many parents unsure about whether and when to disclose ( Tallandini *et al* ., 2016 ). This decision and uncertainties differ depending on whether the treatments were homologous or heterologous, with individuals in the latter category being more reluctant to disclose. This hypothesis correlates with similar studies in couples who adopt, showing that this reluctance to disclose to the child is primarily due to the absence of a biological connection between at least one parent and the child ( Tallandini *et al* ., 2016 ).

In the last two decades, the consensus regarding whether to disclose to the children how they were conceived has changed. Initially, confidentiality was favored, but currently, parents are encouraged to disclose ( Cook *et al* ., 1997 ), with several ethics’ committees having expressed their opinion in favor of disclosure (for example, the Ethics Committee of the American Society of Reproductive Medicine (ASRM) (2004) and the Human Fertilization and Embryology Authority (HFEA) (2008) ( Ethics Committee of the American Society for Reproductive Medicine, 2018 ; Kupka *et al* ., 2014 ). Additionally, laws have been enacted in various countries, ensuring the child’s right to know the identity of donors in the case of heterologous ART treatments ( Portugal, 2018 ). Disclosing brings benefits not only to the family dynamic, by increasing honesty and trust, but also clinically, as it allows children to know their genetic heritage and possess all the necessary information to anticipate potential health problems and prevent consanguinity ( Tallandini *et al* ., 2016 ). It is also relevant to mention the disparity between parents who initially show the desire to disclose and those who actually do so, as a study shows that, although about 52% of parents intend to disclose by the time the child turns 10, only 9% actually do so by the time the child reaches 22 ( Applegarth *et al* ., 2016 ; Tallandini *et al* ., 2016 ).

As previously mentioned, parents’ decision to disclose the method of conception to their children is significantly influenced by whether the treatment was homologous or heterologous. In cases of heterologous treatments, parents often struggle with disclosure, even if they initially plan to tell their children how they were conceived, particularly in the cases of embryo donation, when both parents lack a genetic connection to the child ( MacCallum, 2008 ). This raises questions about whether this difference in parental attitude may be related to the fact that neither individual has a genetic connection to the child and thus fears a greater threat to the parental relationship. The exception occurs in gestational surrogacy cases, possibly due to its relatively easier explanation to children and the gestational carrier’s possible closer relationship with the family, as she is usually a family member or someone close to the family ( Golombok *et al* ., 2006 ). Justifications for non-disclosure often revolve around a desire to protect the child’s privacy or difficulties in finding the right words and timing for disclosure ( Applegarth *et al* ., 2016 ; Tallandini *et al* ., 2016 ).

The timing and manner of disclosure significantly impact the psychological well-being of both parents and children, affecting the child’s perception of this matter. Early disclosure allows children to develop a better understanding of their conception process over time and incorporate the information in a more organic way leading to more comfortable and open conversations within the family ( Applegarth *et al* ., 2016 ). These parents make an early decision about when to disclose, allowing the children to grow up always knowing their conception story ( Applegarth *et al* ., 2016 ). This experience of progressive and continuous dialogue is lost when families postpone this disclosure and increasingly fear this moment ( Applegarth *et al* ., 2016 ). Delaying disclosure often leads to increased parental anxiety, especially as children approach adolescence and adulthood. Families who opt for early disclosure report lower levels of anxiety and experience fewer conflicts, with less frequent and less severe disputes between mothers and children, fostering a more positive parent-child relationship ( Applegarth *et al* ., 2016 ; Brennan D. Peterson *et al* ., 2006). Contrary to fears, disclosure does not seem to have adverse effects and may even positively impact familial dynamics ( Applegarth *et al* ., 2016 ).

There is a study reporting a significant percentage of individuals conceived through gamete donation discovered this origin after a discussion with parents, from someone other than the parents, or ended up discovering it on their own ( Mahlstedt *et al* ., 2010 ; Brennan D. Peterson *et al* ., 2006). This unintentional path to disclosure poses a potential threat to honesty in the parent-child relationship, potentially creating a climate of distrust within the family. In fact, evidence suggests that individuals who discover their conception through third parties show hostile attitudes and greater distrust towards their parents ( Peterson et al., 2006a ; b ). Nevertheless, even though these concerns warrant consideration, studies have shown that children in families who chose not to disclose show no significant differences in psychological well-being compared to those conceived with their parents’ gametes. However, children who have been misled by their parent(s) as to their genetic beginnings may later experience harm should they discover their origins later in life and/or from someone other than a parent. In the age of direct-to-consumer genetic testing, the likelihood that donor-conceived individuals will never discover their method of conception is very low. Therefore, this finding may not reflect the current attitudes and desires of donor-conceived individuals, who increasingly advocate for openness and transparency about their genetic origins. Therefore, it is essential to consider that early and honest disclosure might better support their long-term well-being and potential psychological harm might occur if they find out later in life.

Analyzing the parental relationship between women who engage to the ROPA method and their children, it is far from clear. Some women believe that biological connection reaffirms the social and emotional connection to the child and the “mother role,” which is one of the main reasons they engage to this method over monoparental ones ( Vitule *et al* ., 2015 ). On the one hand, some authors argue that the presence of biological ties adds no additional value, arguing that this relationship is independent of its existence, adding that biological ties should not play any role in the distribution of tasks and authority within parents’ projects ( Di Nucci, 2016 ). On the other hand, studies show that when this biological connection is absent, this fact is noticed by the couple, and although lesbian couples show greater equity in caring for children than heterosexual couples, biological mothers tend to be more concerned with these care tasks in cases of single-parent methods, and children tend to seek the biological mother more when they are hungry and the other mother for play ( Greenfeld, 2005 ).

## THE IMPACT OF ASSISTED REPRODUCTIVE TECHNIQUES (ART) ON CHILDREN BORN AND THEIR DEVELOPMENT

The decision to engage to ART is made by adult individuals, logically without the direct participation or prior consent of the children conceived through these techniques. Nevertheless, these children are directly affected by this choice, as their conception and birth are shaped by this context. When these techniques were introduced, questions were raised about their impact on the children born, both physically and psychologically. While concerns regarding physical development quickly proved unfounded, questions regarding psychological development persisted ( Peterson et al., 2006a ; b ). Thus, it becomes important to reflect on the impact ART has on these children, from their emotional and psychological development to their understanding of their own identity and origin. Table 3 presents a concise overview of the literature’s findings regarding how ART impacts the psychosocial well-being of the children born.

**Table 3:** Psychosocial Impact of Assisted Reproductive Techniques (ART) on the Children Born and their Development

Author, year country	Questions/groups analyzed	Main findings
Zweifel, 2015	Analyze the impact of using donor gametes on child well-being	Children born after heterologous treatments show similar emotional and psychosocial development to naturally conceived ones. Children vary in their desire to know more about their conception and generally accept their parents' decision to use donor gametes.
Gibson *et al* ., 2002 Australia	Analyze and compare the behavior of children, parental stress, and family attitudes in families conceiving through ICSI, IVF, and naturally	Mothers using ICSI and IVF express more protective attitudes, with more concerns about child behavior among those using IVF. However, no differences were observed in the behavior of children in these groups compared to naturally conceived ones.
Carneiro *et al* ., 2022	Evaluate the psychological adjustment of children born through ART	No substantial differences were found between children born through ART (including different treatment types) and naturally conceived ones, both in internalizing and externalizing problems.
Zadeh *et al* ., 2018 United Kingdom	Analyze the perspectives of adolescents conceived through surrogacy or gamete donation about their conception	Most adolescents feel indifferent about their conception but also show interest in the third party involved (gestational carrier or donor).
Golombok *et al* ., 2002 Europe	Analyze the transition to adolescence in children born through ART	Children showed equal levels of development and psychological adjustment.
Jadva & Imrie, 2023 United Kingdom	Study the thoughts and feelings of young adults born through heterologous treatments	40% of young adults feel special about their mode of conception, while the rest are neutral or unconcerned. Most do not frequently discuss the issue, but when they do, they do not encounter problems or constraints.

Analyzing the literature, no significant differences were found in scores of behavioral problems or temperament, both in reports from parents and teachers, being similar in families who engaged to ART and those who conceived spontaneously ( Carneiro *et al* ., 2024 ; Gibson *et al* ., 2002 ; Jadva & Imrie, 2023 ; Brennan D. Peterson *et al* ., 2006). These children show equal interest in school activities and good relationships with peers ( Peterson et al., 2006a ; b ). Furthermore, they are equally confident and exhibit equal levels of verbal aggression. One difference noted, although not consistent throughout the literature, is that children born after ART are less physically aggressive ( Peterson et al., 2006a ; b ). Thus, the attitudes described in previous chapters of greater parental protection in ART parents do not seem to have an adverse impact on the child’s adjustment and development and may even reflect greater attention and sensitivity towards the child, related to this unique path to conception ( Gibson *et al* ., 2002 ).

The transition to adolescence is a critical period for identity and autonomy development, marked by increased biological and genetic awareness. This stage poses unique challenges for individuals conceived through ART, especially those involving gamete or embryo donation or gestational surrogacy. Understanding how these children perceive their conception method during adolescence is crucial. Most adolescents show indifference toward their mode of conception, regardless of the type of ART used ( Zadeh *et al* ., 2018 ).

Analyzing the long-term impact, studies have also been conducted with young adults conceived through ART. Most of them are neutral or unconcerned regarding their mode of conception, with a significant percentage of these individuals (40%) feeling unique due to this particularity in their history ( Jadva & Imrie, 2023 ). Although they report that the method of conception is rarely discussed with others, when it is brought up, it does not cause discomfort or problems for these young adults, indicating that they are well-adjusted and comfortable discussing this matter ( Jadva & Imrie, 2023 ).

Regarding heterologous treatments, children conceived this way show typical emotional and behavioral development, with no significant differences compared to naturally conceived peers in terms of their emotional state, symptoms of hyperactivity or attention deficit, and the quality of relationships they establish with friends and family ( Zweifel, 2015 ). Studies indicate that even during adolescence, these children exhibit similar psychosocial development levels and do not report heightened anxiety or issues related to their conception or birth ( Zadeh *et al* ., 2018 ).

It is understandable that whether they know their origin, especially if conceived through gamete donation, may impact how these children perceive identity and particular developmental issues. Thus, it has been suggested that disclosure and the possibility of discovering more information about the donor and/or establishing contact with them can be an empowering experience and a way for children and young people to feel more in control of these challenges. These children may have varying levels of interest in knowing more about their donor or gestational carrier, with some showing a strong desire for information or even contact ( Zadeh *et al* ., 2018 ; Zweifel, 2015 ). This desire is generally greater in families without a socially recognized father, such as single mothers or same-sex couples. Overall, those who establish contact with donors or gestational carriers report positive experiences, with no negative impact on family relationships observed ( Zadeh *et al* ., 2018 ; Zweifel, 2015 ).

The family configuration in which a child is raised, often resulting from ART, can impact their development, warranting separate analysis. Research indicates that children from various family structures exhibit similar levels of psychological and cognitive development, regardless of parental sexual orientation or family size ( Brandão & Ceschin, 2023 ; Carneiro *et al* ., 2024 ). Heterosexual couples usually engage to ART due to infertility, while same-sex couples or single individuals have different motivations. Most studies find no differences in children’s psychological development across family types, but some note lower adjustment problems in children of same-sex couples compared to those with heterosexual parents ( Carneiro *et al* ., 2024 ). Heterosexual families using donor gametes may experience more adjustment difficulties, possibly due to greater reservation about discussing conception methods ( Carneiro *et al* ., 2024 ). This confidentiality and taboo surrounding infertility and gamete donation may influence the behavioral and emotional adjustment of children in this group. However, there are studies where these differences were not observed. Regarding families with transgender individuals who used ART, they have only recently gained visibility, with no data analyzing them or their children’s development ( Carneiro *et al* ., 2024 ).

A limitation of the findings presented is that they reflect the experiences of individuals who agreed to participate in the studies, meaning these data may tend to represent only well-functioning families who feel comfortable participating and underrepresent families experiencing greater difficulties ( Zadeh *et al* ., 2018 ; Zweifel, 2015 ).

Regarding posthumous reproduction, the effects on the children are not fully known and the literature is sparse ( Polyakov & Rozen, 2023 ). Children born through PAR face unique challenges, largely because one of their parents is not present from the time they are born. Not having that parent, can be highly damaging emotionally and psychologically for a child as they grow; especially if one learns later in life. The long-term psychological consequences of this are unknown ( Bahadur, 2004 ). Within the fertility industry, some experts contend that today there is unduly emphasis on fulfilling potential parents’ wishes and less of a focus upon what may or will be in the interests of these children born as a result ( Bahadur, 2004 ).

## CONCLUSIONS

The detailed analysis of various situations related to ART reveals an emotional and social complexity that marks the lives of individuals seeking these treatments. From the decision to engage to ART to the psychosocial implications of the different options available, each stage of this process show unique challenges and complex ethical questions.

It is not clear that the emotional impact of these techniques is significant, affecting not only the direct beneficiaries but also their interpersonal relationships, especially the marital relationship with the person with whom they share this parenting experience. Anxiety and depression can emerge as natural responses to the challenges faced during treatment, especially in cases where expectations are not met. Additionally, factors such as age, reproductive history, and the availability of emotional support play a crucial role in individuals’ adaptation to these challenging circumstances.

There are also particular situations, such as the use of heterologous treatments, including the ROPA method, and surrogacy, which present additional layers of emotional and ethical complexity, with their own challenges.

Moreover, disparities in health systems and laws of different countries highlight the importance of the social context in the accessibility and impact of ART. The availability of resources, governmental regulation, and cultural norms vary widely, significantly influencing the experience of individuals engaging to ART.

The legal uncertainty and emotional challenges faced by individuals wishing to become parents through surrogacy highlight the urgent need for a more comprehensive and ethical approach in regulating these techniques. It is essential to ensure that all parties involved are protected and that the rights and interests of future parents, pregnant women, and children are duly considered.

The impact of ART on the parenting relationship was also analyzed, concluding that it is complex and multifaceted. Although studies suggest that the use of these techniques does not have direct negative effects on parenting or the relationship established between parents and children, there are important nuances to consider.

The anxiety and depression experienced by future parents during the infertility process and ART treatments can influence the transition to parenthood. Moreover, parents’ concerns about when and how to reveal to their children that they were conceived through ART also affect family dynamics and the trust relationship between parents and children, especially in cases where donor gametes were necessary.

The decision to reveal or not reveal the conception’s origin to the children and the timing of such revelation have significant implications on children’s psychological and emotional development, as well as on family dynamics. While disclosure may generate anxiety in parents, studies suggest that honesty and openness can strengthen family bonds and reduce conflict.

However, there are still gaps in the literature on the psychosocial impact of ART on the parenting relationship, especially in diverse cultural and social contexts. It is crucial to continue investigating this issue to create policies and practices that effectively support parents and promote the well-being of families conceived through ART.

At last, regarding the impact of ART on the development of children conceived through these methods, research over recent decades has extensively explored this issue, suggesting that in most cases, children born via ART do not show significant differences compared to naturally conceived ones in terms of psychological adjustment, academic performance, social interactions, and personal identity. Specific factors such as disclosure of conception origin and access to donor information may affect the emotional and psychological development of ART-conceived children, but overall, they demonstrate resilience to these challenges.

Furthermore, family configuration—whether heterosexual, homosexual, or single-parent—does not seem to significantly impact the development of ART-conceived children. These findings suggest that the quality of the family environment and parental emotional support are more crucial factors than the method of conception itself.

However, it’s important to acknowledge the limitations of existing research, particularly regarding the representativeness of the studied samples. Many studies rely on families who agree to participate, potentially resulting in a biased sample of well-functioning families. Additionally, a significant portion of the research is correlational in nature, which limits the ability to draw causal inferences. Correlational studies can identify associations between variables, but they do not establish cause-and-effect relationships. This is a critical limitation, as many studies cannot conclusively determine whether observed psychological outcomes are directly caused by ART procedures or are influenced by other underlying factors, such as pre-existing mental health conditions, socio-economic status, or stress related to infertility itself. Furthermore, the generalizability of findings can be limited due to varying cultural, legal, and healthcare contexts across different studies.

Therefore, further research is needed to fully understand the impact of ART techniques on child development. Nevertheless, current evidence suggests that ART-conceived children generally have the potential to thrive as much as naturally conceived children. Thus, it’s crucial to continue supporting these families and ensure they have access to resources and services that promote their emotional and psychological well-being over time, ensuring that all children, regardless of their method of conception, have the opportunity to reach their full potential and lead happy and healthy lives.

More than a thousand articles have been published on the emotional well-being of fertility patients and their children in recent years. The inclusion of so many different areas of focus also makes difficult to detail each aspect of the different psychosocial dynamics involved, but one of the authors’ ambitions was, precisely, to try to combine in the same work a general vision and that sought to list the most impactful situations in the point of view of the psychosocial well-being of the various protagonists of medically assisted procreation treatments.

In summary, this review underscores the need for a holistic and compassionate approach to infertility treatment and the provision of ART services. Recognizing and addressing the emotional, ethical, and social challenges faced by beneficiaries of these treatments and the resulting children is essential to ensuring adequate support and a more positive and satisfactory treatment journey. Parenthood extends beyond biological conception and is shaped by a variety of factors, including emotional experiences, cultural contexts, and family dynamics. By better understanding the impact of ART techniques on parenthood, we can provide the necessary support for all families to thrive and grow with love and mutual respect.
